# Decoding Ribosome Stress: How NAT10 Influences Pancreatic Acinar Cell Survival and Cancer Development

**DOI:** 10.1016/j.jcmgh.2025.101516

**Published:** 2025-04-30

**Authors:** Jennifer M. Bailey-Lundberg

**Affiliations:** Department of Pathology, Microbiology and Immunology, The University of Nebraska Medical Center, Omaha, Nebraska; Fred and Pamela Buffett Cancer Center, The University of Nebraska Medical Center, Omaha, Nebraska

Ribosomes, discovered in 1955 by Dr. George Palade, are essential cellular machinery responsible for translating messenger RNAs (mRNAs) into proteins, a fundamental process in all living cells.^1^^,^^2^ Their critical role in protein synthesis makes them highly conserved across species. Although much of the research on ribosome biogenesis (RiBi) and mRNA translation has been limited to in vitro studies or studies in developmental biology related to ribosomopathies, disruptions in RiBi or translation inhibition can lead to cell proliferation arrest.^3^ These disruptions also stabilize p53, a tumor suppressor, through a phenomenon called “nucleolar stress,” underscoring the essential role of ribosome production in cell survival. However, the dynamics of ribosome function in adult cells, particularly in tissue regeneration and cancer, are not well-understood.

In this issue of *Cellular and Molecular Gastroenterology and Hepatology*, Cho et al^4^ explore the role of NAT10, an ATP-dependent RNA N-acetyltransferase that modifies ribosomal RNA (rRNA) and transfer RNA (tRNA) with N4-Acetylcytidine (ac4C).^5^ NAT10 is the only enzyme known to add ac4C to targeted RNAs. Secretory cells, which exhibit high levels of ribosomal proteins, depend heavily on ribosome function for protein synthesis, storage, and release. Yet, the role of NAT10 in these cells remains poorly understood. Acinar cells in the pancreas, which are specialized to synthesize digestive enzymes crucial for breaking down lipids, proteins, and carbohydrates, can also serve as the origin of pancreatic cancer.^6^

Cho et al make a significant contribution by demonstrating that NAT10 plays a critical role in RiBi in pancreatic acinar cells. They show that NAT10 supports both rRNA modification and ribosome structural integrity, particularly through its interaction with RPS6, a small ribosomal subunit protein. The authors generate a cell-specific Nat10 conditional knockout mouse model using CRISPR-Cas9 technology (*Nat10*^*flox/flox*^) and cross it with *CAGG:CreER*^*TM*^ mice to delete Nat10 in various tissues after tamoxifen administration. Loss of Nat10 results in defects in 18S rRNA biogenesis, leading to a reduced 18S/28S rRNA ratio and dysfunction in 40S ribosomal subunit assembly. Notably, the 60S subunit remains unaffected, indicating a specific dependency of 18S rRNA on Nat10 processing.

The investigators further generated *Nat10*^*flox/flox*^*;Mist1*^*CreERT2/+*^ mice (*Nat10*^*Δ/Δ*^*)* to delete *Nat10* specifically in pancreatic acinar cells. In these mice, delayed apoptotic cell death was observed, characterized by significant cellular shrinkage and increased caspase-3 activity 5 to 6 weeks later. This delayed response suggests that, although NAT10 is important for maintaining rRNA ratios and protecting against apoptosis, its loss leads to a prolonged latent period before phenotypic changes manifest. These changes are linked to p53 stabilization, a key stress response regulator, highlighting the role of RiBi stress in activating apoptotic pathways in secretory cells.

Interestingly, deletion of p53 in Nat10-deficient cells rescued acinar cells from apoptosis, but did not restore cellular function. Lineage tracing showed that the *Nat10*^*Δ/Δ*^*;Trp53*^*Δ/Δ*^ double knockout mice exhibited increased nucleolar size and acinar cell proliferation, yet failed to produce amylase, a key enzyme in pancreatic secretory function. These data underscore the critical role of NAT10 in maintaining the highly specialized function of acinar cells.

Cho et al also investigated the role of NAT10 in the development and progression of pancreatic ductal adenocarincoma (PDAC), a topic of increasing interest given the association of elevated NAT10 expression with poor prognosis in pan-cancer cohorts (reviewed in^7^). Zong et al recently published that *NAT10* mRNA and protein expression are elevated in PDAC and NAT10 protein expression was associated with poorer overall survival and PDAC metastasis by increasing AXL expression.^8^ Additionally, Huang et al have shown NAT10 promotes perineural invasion in PDAC through ac4C modification of *ITGB5* mRNA.^9^ In the manuscript by Cho et al, a model with a conditional and inducible *Kras*^*G12D*^ mutation in *Mist1*^*+*^ cells, *Nat10*^*Δ/Δ*^*;Kras*^*G12D*^ mice failed to develop pancreatic intraepithelial neoplasia lesions or invasive PDAC, even though cell cycle re-entry and proliferative activity occurred. This suggests that NAT10 is crucial not only for maintaining acinar cell function but also for the initiation and progression of PDAC. Further experiments with *Nat10*^*Δ/Δ*^*;Kras*^*G12D*^*;Trp53*^*Δ/Δ*^ mice demonstrated that, unlike *Kras*^*G12D*^*;Trp53*^*Δ/Δ*^ mice that developed invasive PDAC, the triple knockout mice did not develop pancreatic intraepithelial neoplasia or PDAC, despite re-entering the cell cycle.

This study provides significant insights into the role of RiBi in specialized, differentiated cells like pancreatic acinar cells in vivo. The use of the Nat10 conditional knockout mouse model reveals that acinar cells are uniquely susceptible to RiBi defects, leading to a delayed, p53-dependent apoptotic response. Importantly, the loss of cellular function, including the breakdown of digestive enzyme cargo, was not rescued by p53 deletion, challenging the conventional idea that p53 stabilization uniformly leads to cell death in response to RiBi defects.

The delayed response to RiBi defects in acinar cells suggests a threshold or tipping point for p53 activation, a scientific concept that warrants further studies. These findings highlight the complexity and significance of RiBi inhibition in vivo, especially in non-dividing, differentiated cells, which contrasts with the rapid cell cycle arrest or senescence typically observed in in vitro models.

This study has important implications for the field of pancreatic cancer research. Although these data show NAT10 and RiBi promote PDAC, divergent studies have been shown in other in vivo model systems. For instance, zebrafish models have shown tumor-suppressive roles for ribosomal proteins in certain lines, indicating that translation defects can precede tumor development.^10^^,^^11^ In the work by Cho et al, although acinar cells typically respond to injury through acinar-to-ductal metaplasia, they failed to undergo this process when RiBi was impaired, suggesting that targeting RiBi could be a novel approach for PDAC prevention and treatment, especially in the context of oncogenic *Kras* mutations and *Trp53* loss.

In summary, the elegant work by Cho et al demonstrates the importance of cell-specific approaches to study RiBi and p53 interactions in maintaining cellular homeostasis, responding to injury, and promoting cancer progression. This research offers valuable therapeutic insights, particularly for pancreatic diseases.
